# The Double-Max method: a novel method for gallbladder epithelial biopsy

**DOI:** 10.1016/j.vgie.2022.07.001

**Published:** 2022-08-20

**Authors:** Shun Fujiwara, Masanori Kobayashi, Kazuo Ohtsuka, Minoru Tanabe, Ryuichi Okamoto

**Affiliations:** 1 Department of Gastroenterology and Hepatology, Tokyo Medical and Dental University (TMDU), Tokyo, Japan; 2 Department of Hepatobiliary and Pancreatic Surgery, Tokyo Medical and Dental University (TMDU), Tokyo, Japan

**Keywords:** ETGBD, endoscopic transpapillary gallbladder drainage, POCS, peroral cholangioscopy

## Abstract

Video 1 A novel biopsy method for gallbladder epithelial biopsy.

A novel biopsy method for gallbladder epithelial biopsy.

## Case Presentation

**Figure 1:**
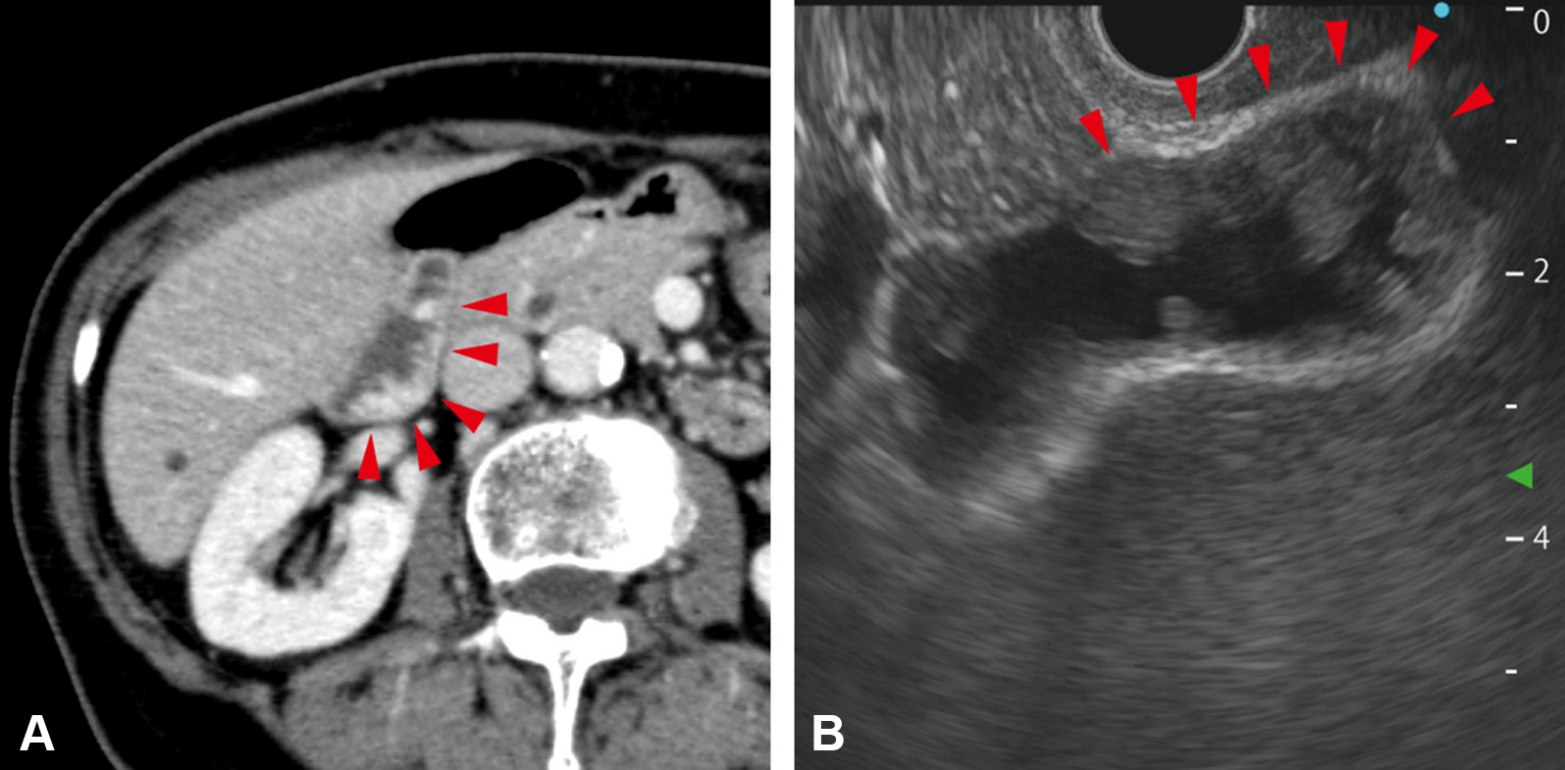
Images of the gallbladder lesion. **A,** Contrast-enhanced CT revealed that there was irregular thickening of the gallbladder wall (*red arrows*), although extramural invasion was not evident. **B,** EUS revealed that there were many mobile polyps larger than 10 mm in the gallbladder. Wall thickening also was observed in a part of the gallbladder wall (*red arrows*), but no extramural invasion was detected.

A 75-year-old woman with no medical history presented to her previous physician complaining of weight loss. The patient was referred to our hospital after abdominal ultrasonography revealed a gallbladder lesion. There was irregular thickening of the gallbladder wall on a contrast-enhanced CT scan (Fig. 1A) and multiple broad-based polyps on EUS (Fig. 1B). Endoscopic retrograde cholangiopancreatography was performed to determine whether the gallbladder lesion was malignant.

## Procedure

**Figure 2:**
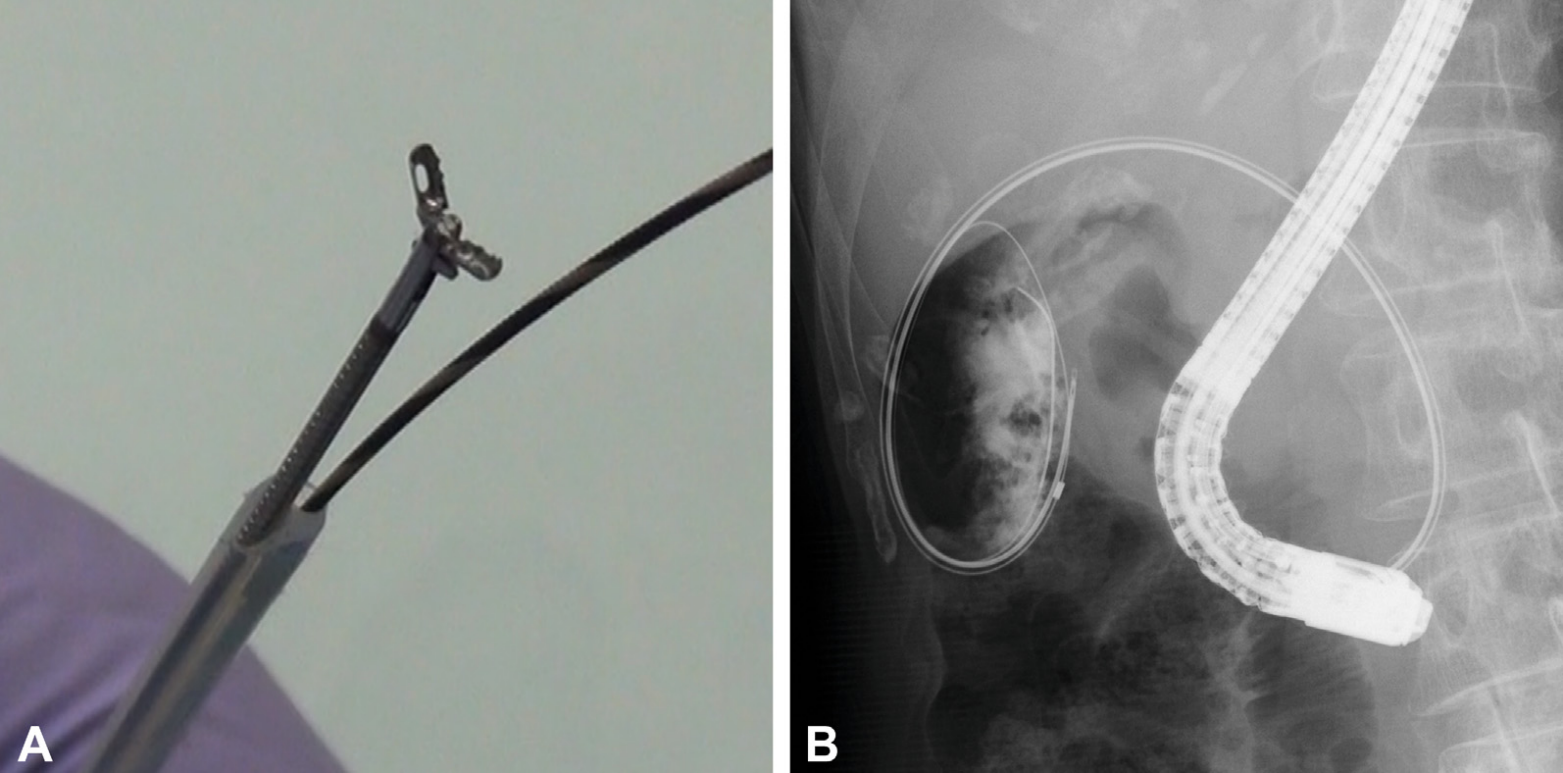
The Double-Max method. **A,** Cytomax II double-lumen biliary cytology brush, with SpyBite Max Biopsy Forceps inserted in the main lumen, in preparation. The tail of the guidewire placed in the gallbladder is fed through the side lumen. **B,** Biopsy forceps can also be used in retroflex position, which allows for the collection of tissue samples from a wide area.

The biopsy method we previously reported using the external sheath of the CytomaxII double-lumen biliary cytology brush (Cook Medical, Bloomington, Ind, USA) was used (Video 1, available online at www.giejournal.org).^1^ We called this the *Double-Max method*, in which SpyBite Max Biopsy Forceps (Boston Scientific, Natick, Mass, USA) were inserted into the main lumen after the brush and the connector were removed. The guidewire (Endoselector, Boston Scientific, Natick, Mass, USA), placed in the gallbladder, was fed through the side lumen (Fig. 2A). The biopsy forceps were guided to the gallbladder along the guidewire using the cytology device. Leaving the cytology device in place, the forceps could be easily removed and reinserted. Additionally, biopsy forceps can be used in retroflex positions to collect tissue samples from a wide area of the gallbladder (Fig. 2B).

## Outcome

**Figure 3:**
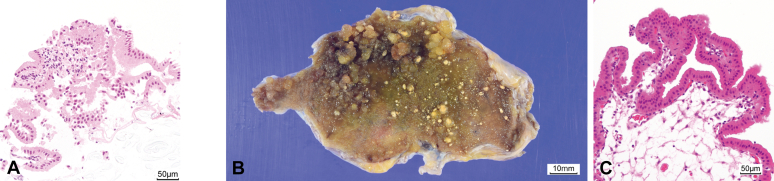
Histological images. **A,** H&E staining of the biopsy specimen taken from the gallbladder showed normal cylindrical epithelium with no evidence of malignancy (H&E, orig. mag. ×100). **B,** Macroscopic image of the surgical specimen showed a large number of yellowish or light brown polyps up to 5 mm at the gallbladder fundus. **C,** H&E staining of the surgical specimen revealed cholesterol polyps with no malignant findings, consistent with the biopsy results (H&E, orig. mag. ×100).

This method provided sufficient sample volume for tissue diagnosis and revealed normal cylindrical gallbladder epithelium with no evidence of malignancy (Fig. 3A). Based on this result, a simple cholecystectomy was performed. The final pathology revealed cholesterol polyps with no malignant findings consistent with the biopsy results (Fig. 3B and C).

## Discussion

Despite advancements in imaging technology, it is still difficult to distinguish benign from malignant gallbladder lesions.^2^ In the case of suspected malignancy, an extended cholecystectomy is performed, but the surgery would be overly invasive if no malignancy is found. It is therefore important to confirm histologic diagnosis before surgery to determine the treatment strategy. In previous studies, histologic diagnosis by EUS-guided FNA and endoscopic transpapillary gallbladder drainage (ETGBD) has been found to be useful.^3^^,^^4^ However, these methods also have risks and limitations. Although EUS-guided FNA has been reported to have a high sensitivity, this procedure is technically difficult and there is a risk of peritoneal dissemination due to bile leakage.^5^ In performing ETGBD, although endoscopic transpapillary cannulation of the gallbladder was successful in 75% to 86.4% of cases, the sensitivity of cytology alone from ETGBD is reported to be 73.3%, which is not sufficient.^6^^,^^7^ Recently, a biopsy of the gallbladder using peroral cholangioscopy (POCS) also has been reported.^8^ POCS offers the advantage of performing a biopsy on the targeted lesion under direct vision, which is thought to provide a high degree of diagnostic certainty. On the other hand, the disadvantages are that it may not pass through the cystic duct and angulated biopsy is difficult. Therefore, we must develop a more reliable and safer diagnostic method for gallbladder diseases.

Although the biopsy forceps used in our method are designed for POCS and are therefore smaller than usual biopsy forceps, they can obtain enough specimen for diagnosis. Our method also permits the biopsy forceps to be used in a retroflex position within the gallbladder, allowing for a greater variety of tissue collection sites. Certainly, our method does not guarantee a biopsy from the targeted lesion, so even if the biopsy is negative, malignancy cannot be completely ruled out. However, this method is expected to improve the diagnostic sensitivity for relatively large lesions such as in our present case, and cases of xanthogranulomatous cholecystitis. Our method, named the Double-Max method, may provide a more reliable and accurate preoperative histologic diagnosis of gallbladder diseases, but further studies comparing with other sampling methods are required.

## Disclosure

*All authors disclosed no financial relationships.*

## Supplementary data

Video 1 A novel biopsy method for gallbladder epithelial biopsy.
